# Diagnostic performance of urinary adipsin as a screening biomarker for pre‐eclampsia

**DOI:** 10.1002/ijgo.70619

**Published:** 2025-10-27

**Authors:** Bismark Opoku Mensah, Ernestina Anim, Linda Ahenkorah Fondjo, Bernard Gyan, Peter Paul Mwinsanga Dapare

**Affiliations:** ^1^ Department of Biological Sciences University of Worcester Worcester UK; ^2^ Department of Nursing All Nations University Koforidua Ghana; ^3^ Department of Molecular Medicine KNUST School of Medicine and Dentistry Kumasi Ghana; ^4^ Department of Medical Diagnostics College of Health and Well‐Being Kintampo Ghana; ^5^ Department of Biomedical Laboratory Science University for Development Studies Tamale Ghana

**Keywords:** blood pressure, diagnostic performance, predictive value, pre‐eclampsia, sensitivity, specificity, urinary adipsin, urine protein

## Abstract

**Objective:**

This study evaluated the diagnostic performance of urinary adipsin as a potential biomarker for pre‐eclampsia screening in a low‐resource setting.

**Methods:**

A case–control study was conducted involving 150 pregnant women classified into three groups: 50 healthy pregnancies, 50 high‐risk pregnancies without pre‐eclampsia, and 50 pregnant women clinically diagnosed with pre‐eclampsia. Urinary adipsin concentration was measured with a commercial sandwich ELISA kit. Linear regression was conducted to examine the relationship between urinary adipsin levels and pre‐eclampsia risk, while receiver operating characteristic (ROC) curve analysis was used to assess the diagnostic performance of urinary adipsin.

**Results:**

Urinary adipsin levels were significantly higher in pre‐eclamptic women compared to healthy controls and the high‐risk group (1258.70 ± 342.88 ng/mg Cr vs. 284.16 ± 24.04 ng/mg Cr and 305.15 ± 36.10 ng/mg Cr; *P* < 0.001). At a cutoff value of 750 ng/mg Cr, urinary adipsin demonstrated a good diagnostic performance, with a sensitivity of 83.6% and a specificity of 88.9%. Combining urinary adipsin with blood pressure measurements significantly improved the predictive performance (*P* < 0.001), with 96.7% sensitivity, 91.0% specificity, and an area under the curve (AUC) of 0.930. The combination of urinary adipsin, urine protein‐to‐creatinine ratio (uPCR), and blood pressure measurement further enhanced specificity (99.7%) with a sensitivity of 90.5% (AUC = 0.909).

**Conclusion:**

Urinary adipsin showed high sensitivity, specificity, and predictive value in diagnosing pre‐eclampsia. This highlights its potential as a non‐invasive biomarker for pre‐eclampsia screening in low‐resource settings. Routine urinary adipsin assay and blood pressure monitoring could enhance early detection strategies and improve maternal and perinatal outcomes.

## 1 INTRODUCTION

Pre‐eclampsia is a life‐threatening hypertensive disorder of pregnancy that contributes significantly to maternal and perinatal mortality worldwide.^1^ Clinically, it is defined as new‐onset hypertension after 20 weeks of gestation together with maternal organ or placental dysfunction and proteinuria.^2^ It affects approximately 10% of pregnancies globally and accounts for nearly 14% of maternal and 10%–25% of perinatal deaths.^3^ In Ghana, the prevalence ranges between 6.55% and 7.30%, making it a significant cause of both maternal and neonatal mortality.^4^

Early identification of at‐risk pregnant women is crucial for effective management and better pregnancy outcomes.^5^ However, current screening methods in Ghana primarily relies on blood pressure measurements, proteinuria assessment, and routine blood chemistry parameters, which are often unreliable and non‐specific. There is a strong need for a one‐stop pre‐eclampsia screening method, as the gold standard 24‐h urinary protein quantification requires two visits and adds inconvenience to women who may have to travel long distances for maternity care.

Recent studies have highlighted the dysregulation of the complement system in the pathogenesis of pre‐eclampsia, with adipsin emerging as a promising biomarker.^6^ Adipsin, a serine protease involved in the alternative complement pathway, plays a role in inflammation and immune regulation.^7^ Elevated adipsin levels have been associated with pre‐eclampsia pathophysiology reflecting complement activation and placental dysfunction.^8^ Compared to serum‐based markers, urinary adipsin offers a non‐invasive and cost‐effective option with potential applicability in resource‐limited settings. This study therefore aimed to evaluate the sensitivity and specificity of urinary adipsin as a potential spot diagnostic biomarker for pre‐eclampsia, as a standalone marker and in combination with spot urine protein and blood pressure measurements.

## 2 MATERIALS AND METHODS

### 2.1 Study design and population

This study employed a case–control design to investigate urinary adipsin levels in pregnant women. It was conducted at the Koforidua Polyclinic in the Eastern Region of Ghana from January 2024 to February 2025. A total of 150 pregnant women were recruited and categorized equally into three distinct groups: healthy controls (group 1), high‐risk pregnancy without pre‐eclampsia (group 2), and clinically diagnosed pre‐eclampsia (group 3). Pre‐eclampsia was defined according to the International Society for the Study of Hypertension in Pregnancy (ISSHP) guidelines as new‐onset hypertension (systolic blood pressure ≥140 mmHg and/or diastolic blood pressure ≥90 mmHg) on two occasions at least 4 h apart after 20 weeks of gestation together with either proteinuria (≥300 mg per 24 h or urine protein‐to‐creatinine ratio [uPCR] ≥30 mg/mmol), or, in the absence of proteinuria, evidence of maternal organ dysfunction. The sample size was estimated using G*Power version 3.1 software, assuming effect size (*f* = 0.25), *α* = 0.05, and 80% power. This resulted in a minimum requirement of 147 participants. Participants were selected using consecutive sampling, with eligible pregnant women recruited consecutively as they presented at the antenatal clinic or maternity unit during the study period.

### 2.2 Ethical approval

The study received ethical approval from the Committee on Human Research, Publications, and Ethics at Kwame Nkrumah University of Science and Technology (CHRPE/AP/1206/24).

### 2.3 Eligibility criteria

Pregnant women aged 18 years or older with gestational ages between 20 and 42 weeks were eligible to participate in the study. The pre‐eclamptic group included both mild and severe cases who had no other comorbidities. High‐risk pregnancy was defined as the absence of clinical pre‐eclampsia at the time of enrolment alongside the presence of one or more of the following: family or personal history of pre‐eclampsia, chronic hypertension (with or without proteinuria), pre‐existing or gestational diabetes mellitus, maternal age ≥35 years, obesity (body mass index [BMI, calculated as weight in kilograms divided by the square of height in meters] ≥30), multiple gestation, renal disease and autoimmune disorders.

### 2.4 Sociodemographic and clinical data collection

A questionnaire was administered to each consented participant to collect sociodemographic data. A detailed medical history was obtained by reviewing medical records.

### 2.5 Anthropometric and blood pressure assessments

Bodyweight and height were measured using an electronic scale (Garmin Index S2 Smart Scale) and a stadiometer (Seca 213, Leicester), respectively. Blood pressure measurements were obtained using a digital sphygmomanometer (Sejoy DBP‐6673B).

### 2.6 Ultrasound examination

All participants underwent transabdominal ultrasound on the same day as urine collection to assess placental location, fetal biometry, and gestational age using the SonoScape S11 Plus ultrasound system (SonoScape Medical Corp., Shenzhen, China). Gestational age was confirmed using first‐trimester ultrasound scans for all participants. The mean gestational age at the time of dating ultrasound was 12.8 ± 1.6 weeks.

### 2.7 Sample collection and processing

Midstream urine samples (5–10 mL) were collected between 9:00 and 11:00 a.m. during routine antenatal visits for both high‐risk and normotensive participants. For the pre‐eclamptic group, samples were taken at the time of diagnosis. All participants were between 20 and 42 weeks of gestation.

Samples were collected by trained nurses, transported to the laboratory within 15–20 min, centrifuged at 342 ***g*** for 10 min, and stored at −80°C. Additionally, venous blood samples were obtained to measure fasting blood glucose (FBG) and glycated hemoglobin (HbA1c) to assess potential influences on adipsin expression related to metabolism and insulin sensitivity.^9^

FBG and HbA1c concentrations were analyzed using the Sysmex BX‐4000 chemistry analyzer (Sysmex and DiaSys Diagnostic Systems, Germany) and the Quo‐Lab HbA1c semi‐automated analyzer (EKF Diagnostics), respectively. Urine concentrations of protein and creatinine were measured using the Roche cobas c311 automated clinical chemistry analyzer (Roche Diagnostics, Germany).

### 2.8 Quantification of urinary adipsin

Urinary adipsin concentrations were measured using a commercial ELISA kit (Abnova Corporation, UK). Assay precision was assessed using low and high concentration quality control samples included in each run. To account for variability in urine dilution, urinary adipsin levels were normalized to urinary creatinine and expressed as nanograms of adipsin per milligram of creatinine (ng/mg Cr).

### 2.9 Statistical analysis

Data analyses were conducted using SPSS version 19 (SPSS Inc., Chicago, IL, USA). One‐way analysis of variance (ANOVA) was used to compare urinary adipsin levels among groups while Pearson correlation examined the relationship between urinary adipsin levels and clinical parameters. Linear regression analyses were performed to evaluate the association between urinary adipsin levels and placenta position, adjusting for BMI, FBG, and HBA1c.

Multivariable logistic regression was used to assess the diagnostic performance of urinary adipsin as a standalone marker and in combination with blood pressure and urine protein‐to‐creatinine ratio. Receiver operating characteristic (ROC) analysis was used to evaluate sensitivity, specificity, and area under the curve (AUC) for each model. Additionally, a decision curve analysis was performed using R to evaluate the clinical benefit across threshold probabilities for adipsin alone, blood pressure (BP) + uPCR, adipsin + BP, adipsin + uPCR, and adipsin + BP + uPCR. A *P* value of less than 0.05 was considered statistically significant.

## 3 RESULTS

### 3.1 Baseline clinical characteristics

The BMI, maternal and gestational ages were similar across all three groups. However, both systolic blood pressure (SBP) and diastolic blood pressure (DBP) were significantly higher in the pre‐eclamptic group compared to both high‐risk and healthy controls (*P* < 0.001 for both comparisons). SBP increased progressively across groups, with the highest values observed in the pre‐eclamptic group (157.86 ± 15.28 mmHg), followed by the high‐risk group (132 ± 10.32 mmHg) and healthy controls (111.13 ± 7.65 mmHg). Similarly, DBP was elevated in both the high‐risk and pre‐eclamptic groups (98.14 ± 7.07 mmHg and 98.44 ± 7.26 mmHg, respectively) compared to healthy controls (70.29 ± 10.75 mmHg) (Table 1).

**TABLE 1:** Clinical characteristics of study participants.

Variables	Group 1 (*n* = 50)	Group 2 (*n* = 50)	Group 3 (*n* = 50)	*P* value
Maternal age (years)	29.11 ± 5.49	29.14 ± 5.02	30.91 ± 5.07	0.440
BMI	27.54 ± 4.03	26.12 ± 4.09	27.50 ± 5.30	0.317
Gestational age (weeks)	26.52 ± 4.13	27.82 ± 4.02	28.47 ± 5.83	0.201
SBP (mmHg)	111.13 ± 7.65	132 ± 10.32	157.86 ± 15.28	<0.001
DBP (mmHg)	70.29 ± 10.75	98.14 ± 7.07	98.44 ± 7.26	<0.001

*Note*: BMI, calculated as weight in kilograms divided by the square of height in meters. Group 1 = healthy controls, group 2 = high‐risk group, group 3 = pre‐eclamptic group.

Abbreviations: BMI, body mass index; DBP, diastolic blood pressure; PE, pre‐eclampsia; SBP, systolic blood pressure.

Fasting blood glucose (FBG) and glycated hemoglobin (HbA1c) levels were significantly higher in pre‐eclamptic women compared to both high‐risk and healthy controls (*P* = 0.009 and *P* < 0.001, respectively). Spot uPCR was also significantly higher in the pre‐eclamptic, and high‐risk groups compared to healthy controls (*P* = 0.003).

Urinary adipsin levels were significantly elevated in the pre‐eclamptic group compared to both high‐risk and healthy controls (*P* < 0.001). Pre‐eclamptic women exhibited markedly higher urinary adipsin levels (1258.70 ± 342.88 ng/mg Cr) compared to high‐risk (305.15 ± 36.10 ng/mg Cr) and healthy pregnant women (284.16 ± 24.04 ng/mg Cr), representing a 4.43 and 4.13‐fold increase compared to the healthy controls and the high‐risk group, respectively (Table 2).

**TABLE 2:** Biochemical characteristics of the study participants.

Variables	Group 1 (*n* = 50)	Group 2 (*n* = 50)	Group 3 (*n* = 50)	*P* value
FBG (mmol/L)	5.40 ± 1.97	5.77 ± 1.02	6.93 ± 2.12	0.009
HbA1c (%)	5.15 ± 1.27	5.38 ± 0.96	6.07 ± 1.99	<0.001
uPCR (mg/g)	152.10 ± 1.07	362.35 ± 2.17	402.67 ± 4.06	0.003
Urine adipsin (ng/mg Cr)	284.16 ± 24.04	305.15 ± 36.10	1258.70 ± 342.88	<0.001

*Note*: Group 1 = healthy controls, group 2 = high‐risk group, group 3 = pre‐eclamptic group.

Abbreviations: FBG, fasting blood glucose; HbA1c, glycated hemoglobin; uPCR, urine protein‐to‐creatinine ratio.

### 3.2 Correlation between urine adipsin levels with placenta location

**FIGURE 1:**
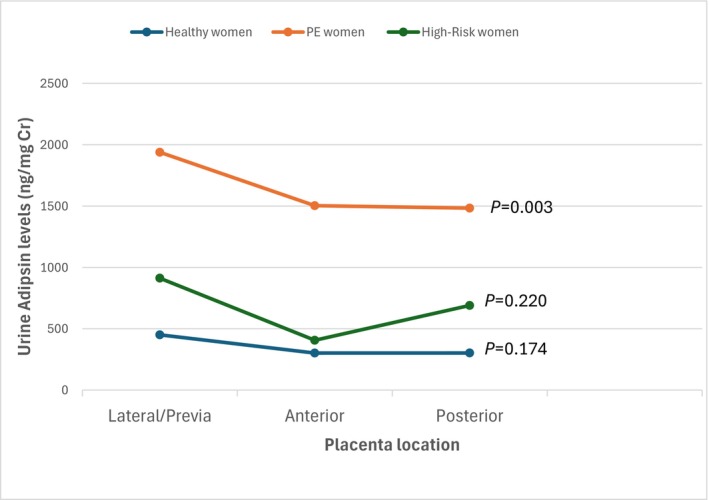
Relationship between urine adipsin levels and placenta location.

A linear regression analysis was used to assess the impact of placenta location on adipsin production, adjusting for BMI, FBG, and HbA1c. The results indicated significant impact of placenta location on urinary adipsin concentrations in pre‐eclamptic pregnancies. Lateral and previa placenta locations were associated with significantly higher adipsin levels (*P* < 0.001), while posterior placenta location showed a significant decrease (*P* = 0.040). Lower adipsin levels were observed in posterior and anterior placenta positions compared to lateral and previa locations (Table 3, Figure 1).

**TABLE 3:** Linear regression analysis of factors associated with adipsin levels.

Variables	Coefficient (*β*)	SE	95% CI	*t*‐value	*P* value
Intercept	284.0	24.04	236.93–331.39	11.82	<0.001
*Placenta location*					
Lateral	78.79	10.00	59.12–98.46	7.88	<0.001
Previa	48.79	8.00	33.07–64.51	6.10	<0.001
Posterior	−20.99	7.00	−34.56–7.30	−3.00	0.040
BMI	1.2	0.50	0.2–2.20	2.40	0.155
FBG (mmol/L)	1.8	0.8	0.00–3.60	2.00	0.071
HbA1c (%)	2.0	0.5	0.80–3.20	43.33	0.018

*Note*: BMI, calculated as weight in kilograms divided by the square of height in meters.

Abbreviations: BMI, body mass index; CI, confidence interval; FBG, fasting blood glucose; HbA1c, glycated hemoglobin; SE, standard error.

### 3.3 Diagnostic performance of urinary adipsin

**FIGURE 2:**
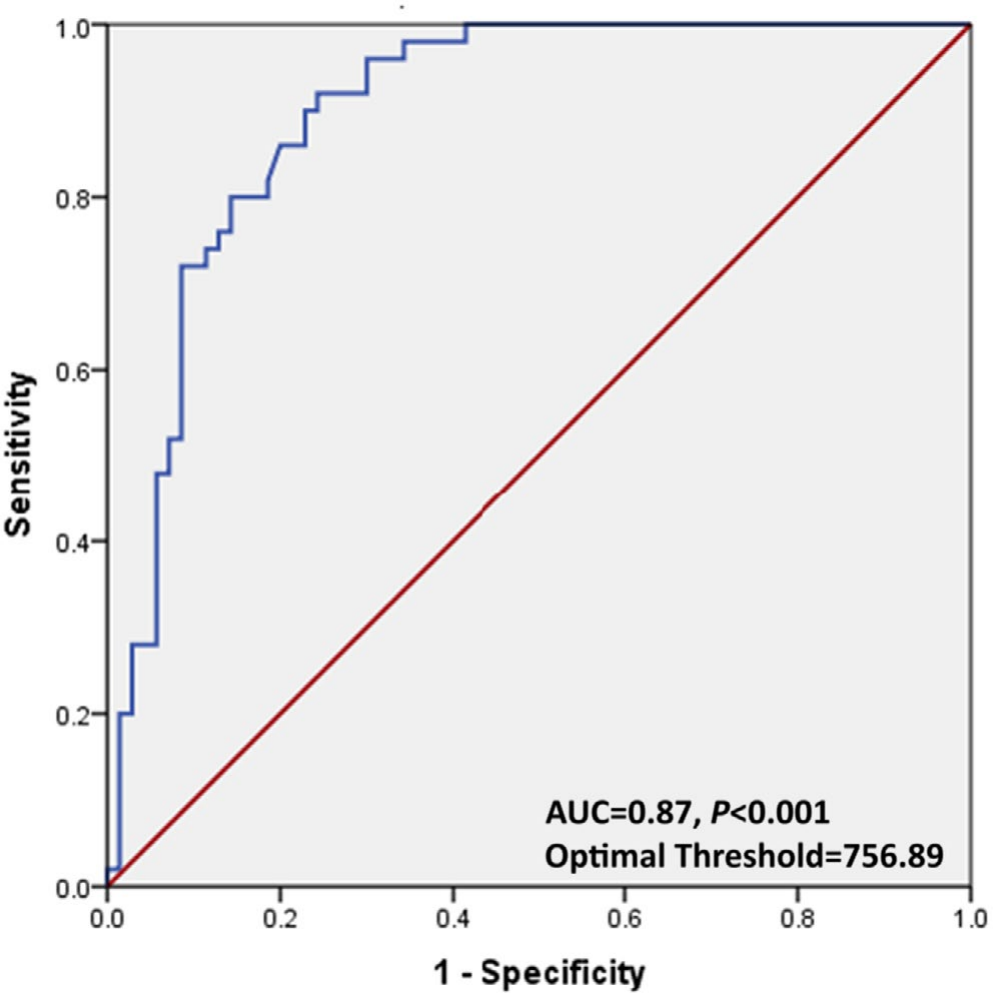
Receiver operating characteristic (ROC) curve for optimum performance of urinary adipsin.

**FIGURE 3:**
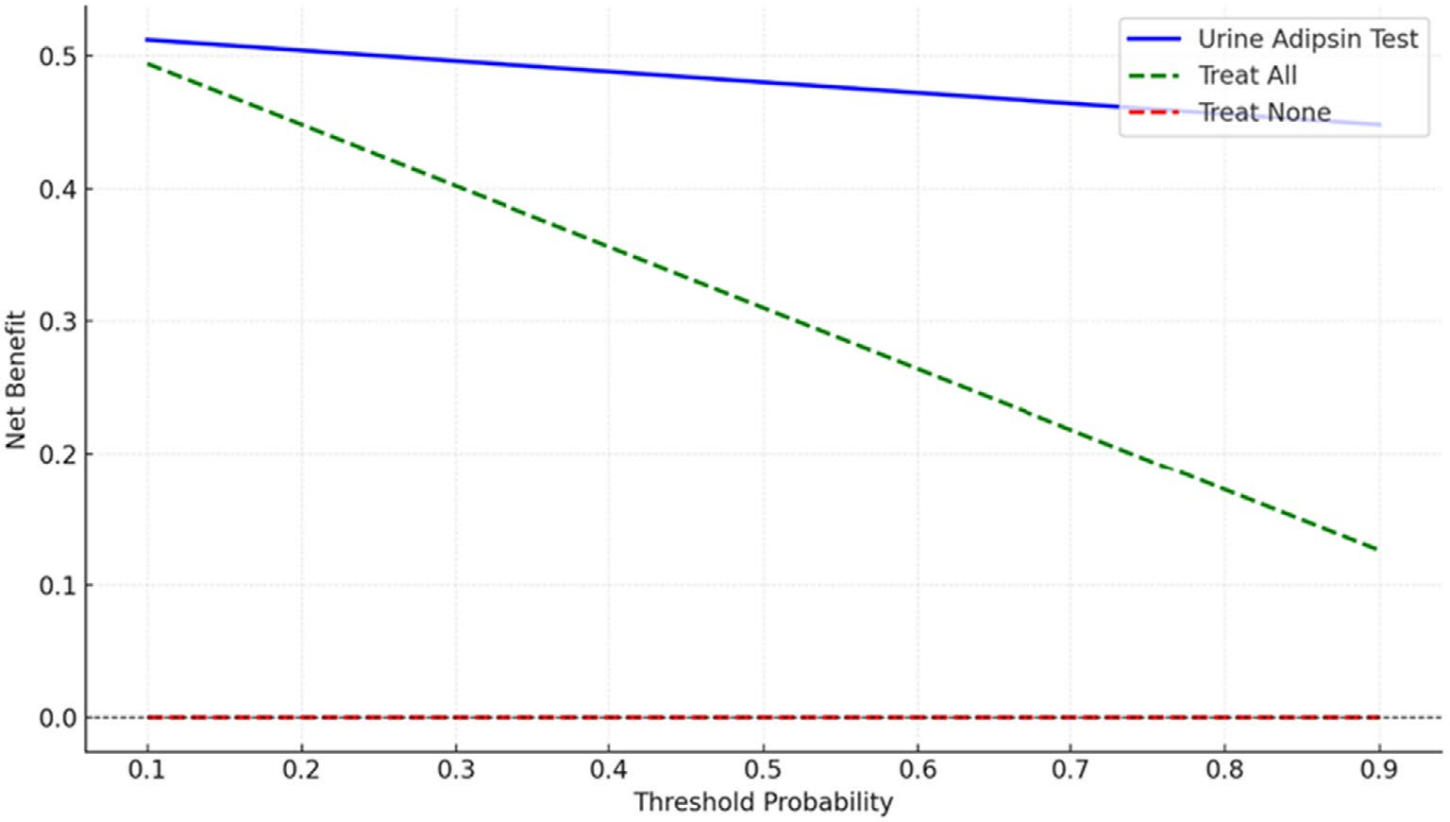
Decision curve analysis for urine adipsin in screening for pre‐eclampsia.

At a threshold of 750 ng/mg Cr, urinary adipsin demonstrated a good diagnostic performance, with a sensitivity of 83.6% and a specificity of 88.9% (Table 4). ROC analysis determined that 756.89 ng/mg Cr was the optimal cutoff value, providing the best balance between sensitivity and specificity (Figure 2). Urinary adipsin outperformed both the “treat all” and “treat none” strategies across a clinically relevant range of threshold probabilities (0.2–0.7) (Figure 3).

**TABLE 4:** Urine adipsin sensitivity and specificity at various cutoff points.

Cutoff (ng/mg Cr)	Sensitivity (%)	Specificity (%)	PPV (%)	NPV (%)	AUC
650	57.1	55.2	31.6	78.0	0.610
700	74.8	36.8	29.9	80.0	0.715
750	83.6	88.9	81.3	90.0	0.870
800	61.9	71.3	43.8	82.8	0.707
850	58.7	52.9	31.1	78.0	0.611

Abbreviations: AUC, area under the curve; NPV, negative predictive value; PPV, positive predictive value.

### 3.4 Diagnostic performance of combinations of urine parameters and blood pressure measurement

When urinary adipsin levels were evaluated alongside urine protein‐to‐creatinine ratio, the combination showed a sensitivity of 76.2%, a specificity of 69.5%, and an AUC of 0.751. The diagnostic performance improved significantly when urinary adipsin was combined with BP measurements (*P* < 0.001), resulting in a sensitivity of 96.7%, a specificity of 91.0%, and an AUC of 0.930.

Further enhancement in performance was observed with a three‐parameter model comprising urinary adipsin, urine protein‐to‐creatinine ratio, and blood pressure. This combination attained the highest specificity (99.7%), but its sensitivity was slightly lower at 90.5% compared to the adipsin and blood pressure combination (*P* < 0.001) (Table 5).

**TABLE 5:** Performance of combination of urinary adipsin, urine protein and blood pressure.

Combinations	Sensitivity (%)	Specificity (%)	PPV (%)	NPV (%)	AUC	*P* value
uPCR + BP (mmHg)	81.4	62.9	48.1	90.8	0.691	0.191
Urine adipsin (ng/mg Cr) + uPCR (mg/g)	76.2	69.5	47.5	89.0	0.751	0.311
Urine adipsin (ng/mg Cr) + BP (mmHg)	96.7	91.0	64.7	97.7	0.930	<0.001
Urine adipsin (ng/mg Cr) + uPCR (mg/g) + BP (mmHg)	90.5	99.7	93.8	96.3	0.909	<0.001

*Note*: Cutoff for urine adipsin ≥756.89 ng/mg Cr, cutoff for uPCR ≥300 mg/g, cutoff for BP: Systolic blood pressure ≥140 mmHg or diastolic blood pressure ≥90 mmHg.

Abbreviations: AUC, area under the curve; BP, blood pressure; NPV, negative predictive value; PPV, positive predictive value; uPCR, urine protein‐to‐creatinine ratio.

**FIGURE 4:**
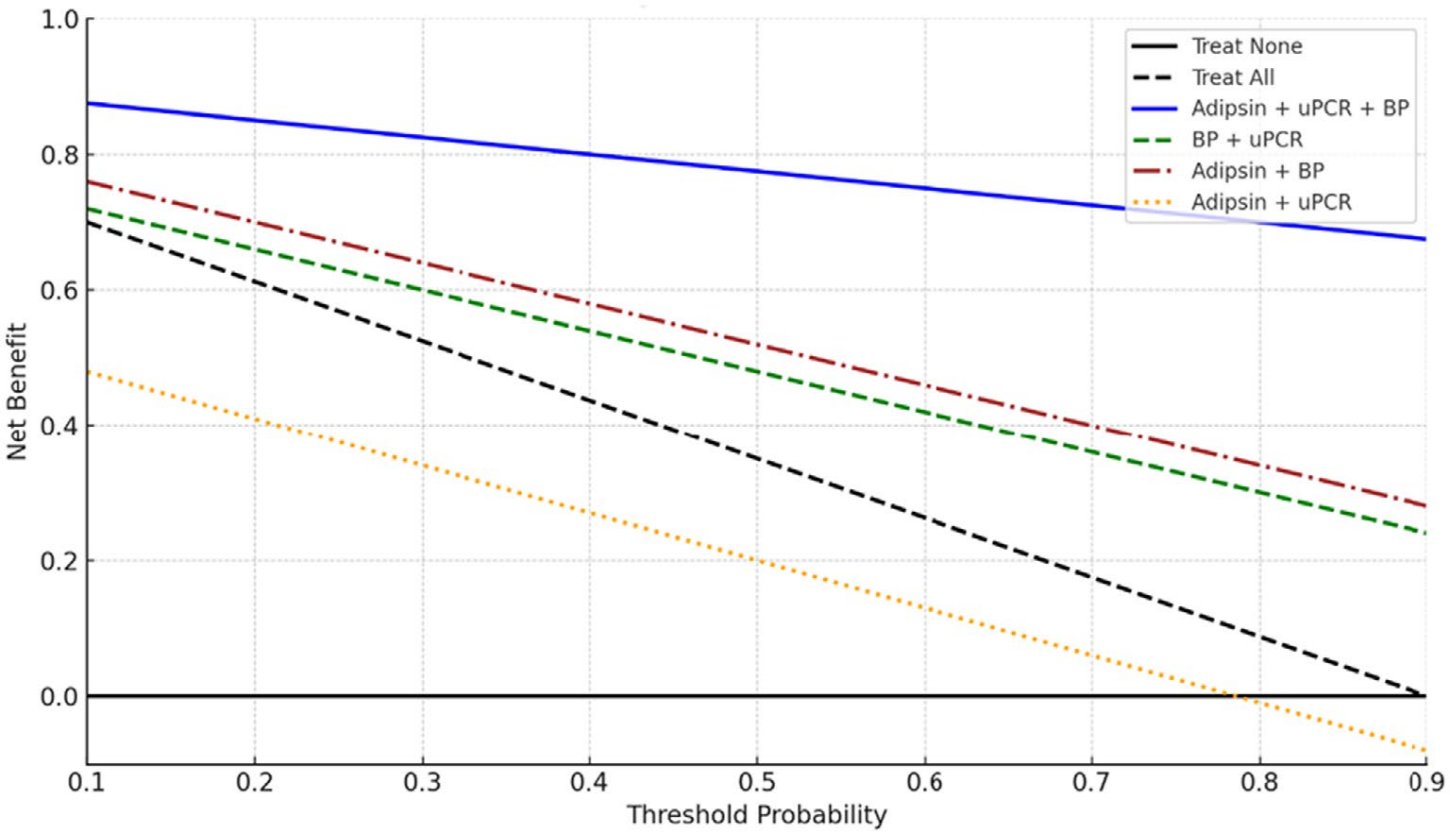
Decision curve analysis of urinary adipsin, urine protein‐to‐creatinine ratio (uPCR) and blood pressure combinations for optimizing pre‐eclampsia screening.

The combination of urinary adipsin, uPCR, and BP provided the highest net benefit across nearly all threshold probabilities when compared to the “treat all” or “treat none” strategies. The BP + uPCR and adipsin + BP models also showed appreciable net benefit. In contrast, the adipsin + uPCR model yielded lower net benefit than the “treat all” strategy (Figure 4).

## 4 DISCUSSION

This study recruited 150 pregnant women to evaluate urinary adipsin as a screening biomarker in low‐resource settings. Participants were divided into three groups: 50 healthy controls (group 1), 50 high‐risk women without pre‐eclampsia (group 2), and 50 women with pre‐eclampsia (group 3).

Maternal age, BMI and gestational age were similar across all groups, minimizing confounding factors as reported by Howes et al.^10^ Our study found that women with pre‐eclampsia had significantly higher blood pressure and uPCR (*P* < 0.001), consistent with previous studies that indicate hypertension and proteinuria are indicators of pre‐eclampsia.^11^, ^12^ This highlights the importance of routine blood pressure and urine protein monitoring during antenatal visits.^13^, ^14^

Urinary adipsin levels were significantly higher in pre‐eclamptic women compared to both healthy controls and the high‐risk group (Table 2). This suggests that urinary adipsin could serve as a potential biomarker for pre‐eclampsia. This is consistent with the study by Wang et al.^15^ and supports the hypothesis that adipsin may play an important role in the pathophysiology of pre‐eclampsia.^16^, ^17^

This study found a significant association between placental location and urinary adipsin levels in pre‐eclamptic pregnancies, with higher levels in lateral and previa placentas. This suggests that placenta orientation may influence function,^18^, ^19^ possibly through altered uteroplacental blood flow and nutrient exchange.^20^ Lateral and previa placenta placements may impair oxygen or nutrient delivery,^21^ potentially affecting the synthesis or secretion of adipsin. This finding supports evidence that abnormal placental development contributes to pre‐eclampsia severity.^22^, ^23^

While our findings suggest an association between placenta orientation and adipsin secretion, orientation was assessed only once in mid‐ to late pregnancy and may not reflect disease onset or biomarker release. Thus, causality cannot be inferred, and longitudinal studies with serial ultrasound and biomarker measurements are needed to elucidate this relationship further.

At a cutoff value of 750 ng/mg Cr and an optimal threshold of 756.89 ng/mg Cr, urinary adipsin showed a clinically relevant accuracy, with sensitivity and specificity of 83.6% and 88.9%, respectively. In contrast, Wang et al. reported higher sensitivity (93.2%) and specificity (98.8%) using rapid test kits,^15^ likely reflecting differences in population, study design, cutoffs, and methodology.

Decision curve analysis showed that adding urinary adipsin to antenatal screening provided clinical benefit over “treat all” or “treat none” strategies. Adipsin performed well across a wide range of thresholds, and models combining it with BP and uPCR consistently offered the greatest net benefit.

The combination of adipsin, uPCR, and BP yielded the greatest net benefit. The BP + uPCR model provided a notable net benefit and aligns with current standard practices. However, its performance was lower than that of the adipsin‐inclusive triple model, suggesting that adding urinary adipsin enhances the existing screening tools. Similarly, the adipsin + BP model demonstrated improved net benefit relative to the “treat none” strategy and outperformed the adipsin + uPCR model, indicating that blood pressure remains a strong anchoring variable in risk prediction models for pre‐eclampsia.

Conversely, the adipsin + uPCR model underperformed, suggesting that without BP measurement, this combination may be insufficient for effective clinical decision‐making. These findings highlight the importance of multiparametric screening, with adipsin serving best as a complementary marker, particularly in low‐resource settings where 24‐h urine collection may not be feasible.

Combining urinary adipsin with uPCR resulted in moderate diagnostic utility (76.2% sensitivity, 69.5% specificity, AUC 0.751), consistent with findings from Bartel et al.,^14^ highlighting the limitations of using proteinuria as a sole diagnostic criterion for pre‐eclampsia.^14^ Urinary adipsin could offer additional value in a one‐stop screening model in settings where 24‐h urine collections or advanced laboratory testing are impractical.

When urinary adipsin was paired with blood pressure measurement, the diagnostic performance significantly improved (sensitivity 93.7%, specificity 80.0%, AUC 0.930). While UPCR and blood pressure alone provided good sensitivity (93.7%) and specificity (85.6%), the inclusion of urinary adipsin increased the specificity to 99.7% while maintaining high sensitivity (90.5%). This incremental improvement is clinically relevant, particularly in minimizing false positives in settings where overdiagnosis can lead to unnecessary interventions. This indicates the synergy between urinary adipsin, reflective of inflammatory and complement system activation,^24^ and elevated blood pressure, highlighting the multi‐pathway nature of pre‐eclampsia.^25^ Additionally, combining urinary adipsin, uPCR and blood pressure achieved an AUC of 0.909, with high specificity (99.7%), demonstrating its potential for minimizing false positives. The observed AUC increase from 0.875 (BP + UPCR) to 0.909 (BP + UPCR + adipsin) supports the additive diagnostic value of urinary adipsin.

Despite these promising results, this study is not without limitations. The findings are based on a single‐center cohort, potentially limiting the generalizability of the results. Future research employing larger, multicenter cohorts and longitudinal study designs are needed to understand the changes in urinary adipsin levels over the course of pregnancy.

## 5 CONCLUSION

This study highlights the potential of urinary adipsin as a non‐invasive biomarker for detection of pre‐eclampsia. Its correlation with hallmark clinical features such as proteinuria and hypertension emphasize its relevance in pre‐eclampsia pathophysiology.

## AUTHOR CONTRIBUTIONS

BOM, EA, LAF, BG and PPD contributed to conceptualization of the research idea, acquisition, analysis and interpretation of data. All authors contributed to drafting of manuscript and approved the final manuscript.

## FUNDING INFORMATION

This study did not receive any form of funding from commercial or public agencies.

## CONFLICT OF INTEREST STATEMENT

The authors declare no conflict of interest.

## Data Availability

The data that support the findings of this study are available from the corresponding author upon reasonable request.
